# Electronic Synergy in Anti-Sandwich Dual-Atom Catalysts on BC_3_ for Efficient Hydrogen Evolution

**DOI:** 10.3390/nano16150930

**Published:** 2026-07-28

**Authors:** Yusong Weng, Wentao Liang, Xuan Chen, Xuefei Liu

**Affiliations:** 1 School of Physics and Electronic Science, Guizhou Normal University, Guiyang 550025, China; 2 School of Integrated Circuit, Guizhou Normal University, Guiyang 550025, China

**Keywords:** synergistic mechanisms, anti-sandwich dual-atom catalysts, HER, BC_3_

## Abstract

The development of efficient electrocatalysts for hydrogen generation is crucial for advancing clean and renewable energy technologies, yet the cooperative behavior governing dual-atom catalytic sites remains poorly clarified. In this study, an anti-sandwich dual-atom architecture supported on a BC_3_ monolayer is examined through comprehensive first-principles modeling. By jointly assessing hydrogen adsorption thermodynamics, metal anchoring strength, and dynamic stability under elevated-temperature ab initio molecular dynamics conditions, two structurally resilient configurations with nearly ideal hydrogen binding characteristics are identified. Detailed orbital-resolved electronic analyses, incorporating projected electronic states, bonding population evaluation, and charge redistribution visualization, indicate that hydrogen adsorption and reaction activity are predominantly controlled by interatomic electronic coupling and orbital hybridization within the dual-atom centers. These insights reveal the electronic essence of dual-atom synergy and establish a transferable design principle for developing high-efficiency anti-sandwich catalysts for hydrogen evolution.

## 1. Introduction

The transition toward a hydrogen-based energy economy has stimulated intensive interest in electrocatalytic hydrogen evolution as a sustainable pathway for clean energy conversion [1,2,3]. Achieving high catalytic efficiency while maintaining structural robustness remains a central challenge in catalyst design [4]. Although platinum-group metals exhibit excellent hydrogen evolution reaction (HER) activity, their scarcity and high cost severely limit large-scale applications, motivating the exploration of earth-abundant alternatives [5,6,7]. In this context, atomic-scale catalyst engineering has emerged as a powerful strategy to maximize metal utilization and tailor reaction energetics [8,9,10]. Among these approaches, dual-atom catalysts (DACs) represent a particularly promising class, as they bridge the gap between single-atom and nanoparticle catalysts by introducing metal–metal cooperation at the atomic level [11,12,13]. However, despite growing experimental and theoretical efforts, the fundamental electronic origin of dual-atom synergy and its role in governing HER activity remain insufficiently understood.

Previous studies have demonstrated that although single-atom catalysts (SACs) possess unique advantages arising from their atomic dispersion [14,15,16], their intrinsically low metal loading and isolated active sites impose fundamental limitations on further performance enhancement [17]. In contrast, DACs, as an extension of SACs, have attracted increasing attention in recent years [18,19,20]. By constructing transition-metal–nonmetal (TM–NM) dual-atom active sites, such catalysts achieve high metal utilization while modulating reaction kinetics through strong metal–nonmetal electronic interactions, with the transition-metal atom serving as the primary active center and enabling tunable structure–activity relationships [21,22,23]. By merging the strengths of homogeneous and heterogeneous catalysis, these systems exploit the cooperative effects between the transition-metal and nonmetal centers, which significantly lower the activation barriers of key rate-limiting steps, ultimately achieving superior performance in practical applications, including proton exchange membrane electrolyzers [24,25,26]. Consequently, catalyst design strategies based on dual-atom synergy have become a major research frontier for improving HER efficiency [27,28,29].

Researchers have devoted considerable attention to understanding the synergistic interactions in DACs, paving the way for the establishment of sophisticated, multi-level analytical paradigms. A representative investigation by Qi et al. [30] demonstrated that Fe–Mo dual-atom sites synergistically catalyze O–O bond formation, thereby promoting oxygen electrocatalysis, and further elucidated the structural evolution and reaction mechanisms of dual-atom catalysts under operando electrochemical conditions. Zhang et al. [31] proposed a multidimensional catalyst design strategy spanning from atomic precision to molecular confinement, highlighting that advanced electrocatalyst design should move beyond simply increasing the number of active sites. Instead, their work underscores the decisive roles of spatial matching and geometric configuration alignment in governing reaction pathways and efficiencies in complex electrocatalytic processes; Wang et al. [32] and co-workers integrated aberration-corrected transmission electron microscopy with first-principles calculations to clarify the role of interfacial charge transfer in regulating HER pathways on Ni–Ru codoped VS_2_ nanosheets. While these studies provide valuable insights through complementary experimental and theoretical approaches, the inherent complexity of DACs, limitations of characterization techniques, and high computational costs have restricted most investigations to specific material systems, hindering the development of broadly applicable theoretical frameworks [33].

Unlike conventional dual-atom catalysts where two metal atoms are directly anchored on the substrate, our proposed “anti-sandwich” architecture consists of one transition-metal (TM) atom and one nonmetal (NM) atom co-anchored on the BC_3_ surface. The TM atom serves as the primary catalytic site, while the neighboring NM atom acts as an electronic modulator, forming a TM–NM–substrate configuration that we term “anti-sandwich” to distinguish it from the symmetric metal–metal dual sites. While Mo- and W-based single-atom catalysts have demonstrated excellent HER performance in previous studies, the present work focuses on 3d transition metals, which exhibit stronger d–p hybridization with nonmetal atoms. Extension of the anti-sandwich design to 4d/5d metals such as Mo and W is an interesting direction for future work.

The BC_3_ monolayer was selected as the substrate because (i) its boron-doped honeycomb lattice provides moderate electronegativity to anchor metal atoms without over-binding, which is critical for maintaining moderate HER activity; (ii) compared with C_3_N_4_ or g-C_3_N_4_, BC_3_ offers superior electrical conductivity, facilitating efficient charge transfer during electrocatalysis; and (iii) the B-C hexagons provide well-defined bridging sites for dual-atom immobilization.

In this work, an anti-sandwich TMNM⊥BC_3_ configuration (where the symbol “⊥” denotes the perpendicular anchoring of the TM and NM atoms on the BC_3_ surface, indicating that the dual-atom pair is adsorbed vertically on top of the substrate, as opposed to being incorporated into the lattice) is constructed by anchoring a transition-metal atom and a neighboring nonmetal atom onto a BC_3_ substrate, with the aim of exploiting cooperative interactions within heteroatomic dual-atom catalysts. First-principles calculations demonstrate that this heteronuclear architecture exhibits excellent structural stability, favorable hydrogen adsorption thermodynamics, and enhanced catalytic activity, highlighting its potential as an efficient electrocatalyst. Nevertheless, conventional descriptors based on electronic localization fail to adequately capture the cooperative effects arising from metal–nonmetal coupling. To address this challenge, systematic electronic-structure analyses, including projected density of states (DOS), crystal orbital Hamilton population (COHP), and charge density difference calculations, are performed to elucidate the nature of electronic interactions between the metal and nonmetal sites. The results reveal that orbital hybridization and electronic redistribution between the two heteroatoms play a crucial role in modulating hydrogen adsorption free energy and catalytic performance. These findings uncover the microscopic origin of synergistic effects in heteroatomic dual-atom systems and provide an electronic-structure-guided strategy for the rational design of high-performance anti-sandwich HER catalysts.

## 2. Methods

The geometric structures and initial configurations of the TMNM⊥BC_3_ systems were constructed using the Device Studio program. Density functional theory (DFT) calculations were primarily performed using the Vienna Ab initio Simulation Package (VASP 6.3.2) [34,35,36]. The electronic exchange–correlation interactions were described within the generalized gradient approximation (GGA) using the Perdew–Burke–Ernzerhof (PBE) functional [37,38], while the interaction between ionic cores and valence electrons was treated using the projector augmented-wave (PAW) method [39].

All atomic structures were fully relaxed until the residual force on each atom was less than 0.03 eV Å^−1^, and the electronic self-consistent convergence criterion was set to 1 × 10^−5^ eV. A plane-wave kinetic energy cutoff of 400 eV was employed throughout the calculations. The Brillouin zone was sampled using a 3 × 3 × 1 Monkhorst–Pack k-point mesh. A vacuum layer of 21 Å was introduced perpendicular to the BC_3_ surface to eliminate interactions between neighboring periodic images. Long-range van der Waals interactions were included using the DFT-D3 correction method [40]. For transition-metal-containing systems, the DFT+U approach was adopted to account for localized electron correlation effects, and all calculations were performed with spin polarization [41,42]. The DFT+U approach was employed to correct the self-interaction error inherent in standard DFT for transition-metal-containing systems. For Ti- and Ni-based systems, standard DFT (PBE) without U typically over-delocalizes the d electrons, which can lead to an incorrect description of the electronic structure and, consequently, inaccurate adsorption energies [42]. The U values for Ti and Ni were adopted from established literature [41,42] and have been widely validated for similar transition-metal systems.

To simulate electrochemical environments, solvent effects were incorporated using an implicit solvation model with water as the dielectric medium ε=78.4 [43,44]. The implicit solvation calculations were performed using the VASPsol implementation [43,44] within VASP, which treats the solvent as a continuum dielectric medium. The relative permittivity of water was set to ε = 78.4, corresponding to the default value for aqueous solution [43,44]. This approach accounts for the dielectric screening effects of the solvent on the electrostatic interactions at the solid–liquid interface, providing a computationally efficient description of the electrochemical environment.

To further investigate the electronic characteristics and structural stability of the optimized catalysts, projected density of states (PDOS) analyses, Bader charge calculations, and ab initio molecular dynamics (AIMD) simulations were carried out using the DS-PAW software package (version 2023B) [36]. The PDOS and Bader analyses were employed to elucidate the electronic interactions and charge-transfer behavior between the dual-metal active sites and the BC_3_ substrate. AIMD simulations were performed within the canonical (NVT) ensemble using a Nosé–Hoover thermostat at 300 K to evaluate the thermal stability of the catalyst systems.

When operated under acidic conditions, the hydrogen evolution process involves multiple elementary reactions, which are generally categorized into the Volmer, Heyrovský, and Tafel mechanisms, as described by Equations (1)–(3) [45].

(1) H++e−→H*

(2) H*+H++e−→H2

(3) H*+H*→H2

The thermodynamic favorability of hydrogen binding was quantified by calculating the free energy change associated with adsorbed hydrogen within the computational hydrogen electrode(CHE) framework [46,47]:

(4) $${∆G}_{H*}={∆E}_{H}+{∆E}_{ZPE}-T∆S$$

where $\Delta E_{H}$ is the hydrogen adsorption energy obtained from DFT calculations, ${∆E}_{ZPE}$ is the zero-point energy correction, and TΔS represents the entropic contribution at 298.15 K. The hydrogen adsorption energy was calculated as [47]:

(5) $$\Delta E_{H}=E_{H}-E_{slab}-\frac{1}{2}E_{H_{2}}$$

A ${∆G}_{H*}$ value approaching zero is considered indicative of optimal HER activity.

The exchange current density (i_0_) was estimated using [48]:

(6) $$i_{0}=-ek_{0}\frac{1}{1+\exp\left(\frac{\left|\Delta G_{H}\right|}{K_{B}T}\right)}$$

where k_0_ is the reaction rate constant and $K_{B}$ is the Boltzmann constant. Larger i_0_ values correspond to higher intrinsic electrochemical activity. To assess the thermodynamic stability of the dual-atom catalysts, the binding energy ($E_{b}$) was calculated as [49]:

(7) $$E_{b}=E_{{TMTM}^{\prime }⊥BC_{3}}-E_{BC_{3}}-E_{TM\left(atom\right)}-E_{TM\prime \left(atom\right)}$$

More negative $E_{b}$ values indicate stronger metal–substrate interactions and enhanced structural stability.

The synergistic effects in DACs were examined through comprehensive electronic-structure characterization, involving density of states, crystal orbital Hamilton population, and differential charge density analyses. Electronic structure analyses were explicitly focused on adsorption sites and adsorbates to directly capture the electronic origin of dual-atom synergy. These analyses reveal how orbital hybridization and electronic coupling between metal and nonmetal sites regulate hydrogen adsorption energetics and catalytic activity, thereby establishing an intrinsic correlation between structural stability and catalytic performance.

## 3. Results and Discussion

**Figure 1:**
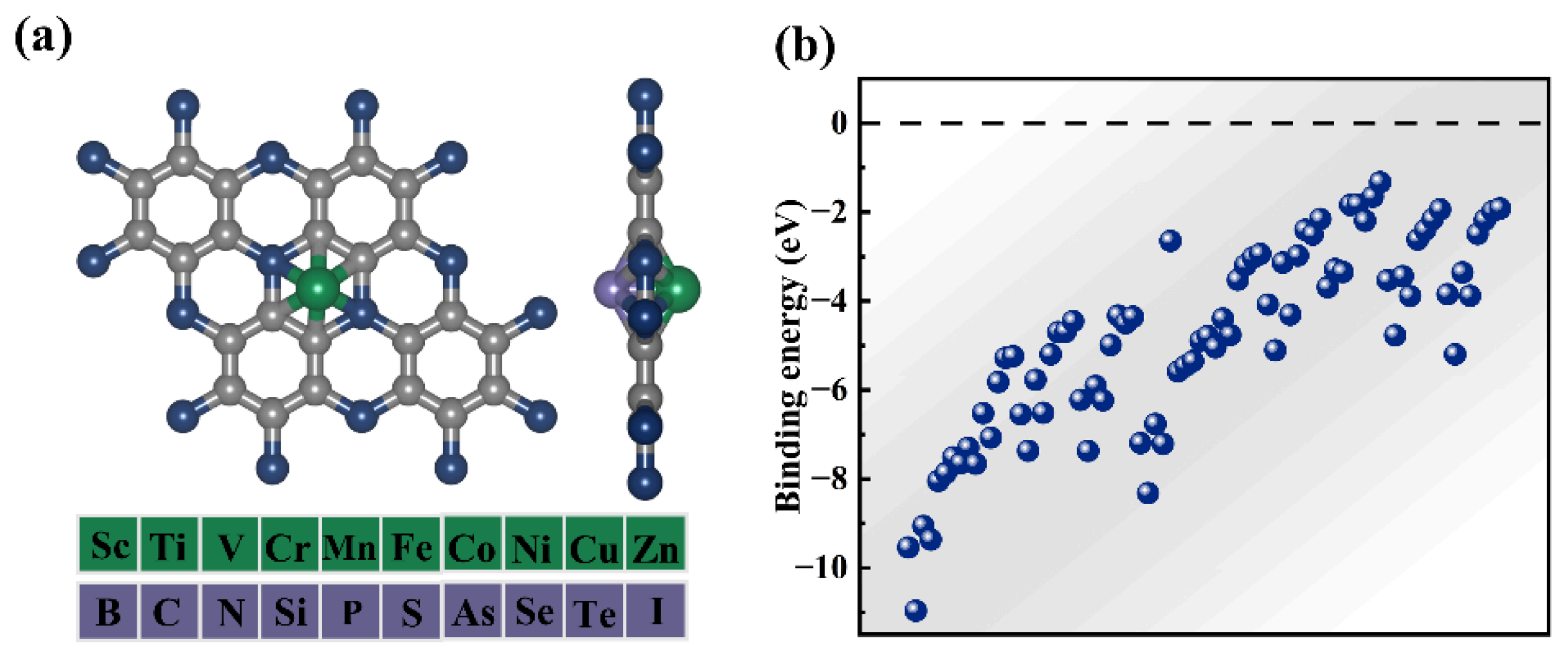
(**a**) Anti-sandwich configuration of dual-atom catalysts, consisting of one TM atom (blue) and one NM atom (purple) co-anchored on the BC_3_ substrate, distinguished from conventional TM–TM dual-atom sites. (**b**) Scatter plot showing the distribution of binding energy.

In this work, an anti-sandwich dual-atom catalytic system (TMNM⊥BC_3_) was constructed using BC_3_ as the supporting substrate (Figure 1a). Fourth-period transition metals (Sc, Ti, V, Cr, Mn, Fe, Co, Ni, Cu, and Zn) and main-group nonmetal elements (B, C, N, Si, P, S, As, Se, Te, and I) were independently anchored at the TM and NM sites during the initial screening stage. The calculations show that TiC⊥BC_3_ exhibits an ultralow hydrogen adsorption Gibbs free energy of only 0.03 eV. This result directly highlights the effectiveness of dual-atom cooperation on the BC_3_ substrate. During model construction, 21 unstable configurations that failed to meet convergence criteria were excluded. As a result, 79 well-converged structures were retained for subsequent catalytic evaluation.

### 3.1. The Catalytic Activity and Stability of TMNM⊥BC_3_

**Figure 2:**
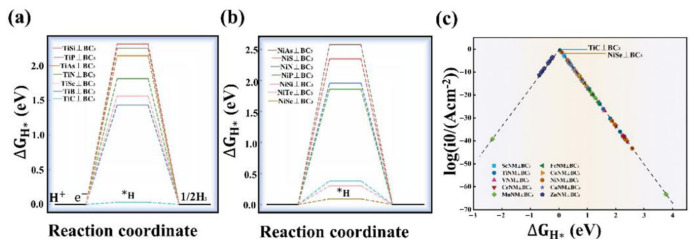
(**a**,**b**) Comparison of hydrogen adsorption thermodynamics and binding stability for the TMNM⊥BC_3_ catalyst series. (**c**) Dependence of the exchange current density on hydrogen adsorption free energy, illustrating the activity trends across different dual-atom configurations. The asterisk denotes a surface adsorption site; *H represents adsorbed hydrogen.

The catalytic activity for the HER was evaluated using the Gibbs free energy of ${∆G}_{H*}$. Values of |${∆G}_{H*}$| lower than 0.09 eV, which corresponds to the activity of commercial Pt catalysts, are generally regarded as indicative of highly efficient hydrogen evolution. This benchmark value is adopted from literature results obtained at the same level of theory (PBE + D3) for the Pt(111) surface. As summarized in Table S1 in the Supplementary Materials, TiC⊥BC_3_ and NiSe⊥BC_3_ stand out among all TMNM⊥BC_3_ configurations (Figure 2a,b). In particular, TiC⊥BC_3_ exhibits a ${∆G}_{H*}$ value very close to the thermoneutral condition, reaching 0.03 eV. To further validate this trend, a quantitative relationship between ${∆G}_{H*}$ and the exchange current density (i_0_) was established (Figure 2c). The predicted optimal catalysts are located near the apex of the volcano plot. Importantly, these results are fully consistent with the ${∆G}_{H*}$-based screening. This dual-validation strategy substantially enhances the reliability of the DFT predictions. It also confirms TiC⊥BC_3_ and NiSe⊥BC_3_ as promising HER catalysts, satisfying both activity descriptors simultaneously. Among the 79 successfully converged TMNM⊥BC_3_ configurations, only TiC⊥BC_3_ and NiSe⊥BC_3_ exhibit |${∆G}_{H*}$| values below 0.10 eV (0.03 and 0.09 eV, respectively), while the remaining candidates show either too weak or too strong hydrogen binding (Table S1). This statistical distribution highlights the exceptional nature of these two combinations and validates the effectiveness of our high-throughput screening strategy.

**Figure 3:**
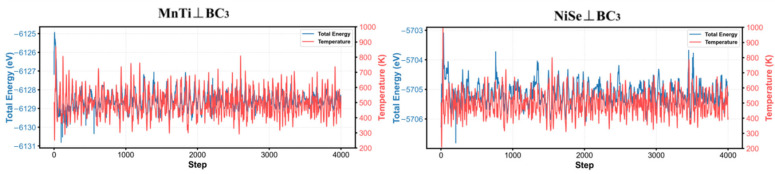
AIMD simulation results for MnTi⊥BC_3_ (**left**) and NiSe⊥BC_3_ (**right**).

The structural stability of the TMNM⊥BC_3_ systems was evaluated using a multi-dimensional approach. Binding energy calculations and ab initio molecular dynamics simulations were combined to probe both thermodynamic and dynamic stability (Figure 1b; Table S2 in the Supplementary Materials). All dual-atom configurations exhibit negative binding energies, indicating strong metal–substrate interactions. For the representative systems NiSe⊥BC_3_ and MnTi⊥BC_3_, additional AIMD simulations were performed at 500 K for 4 ps (Figure 3). As shown in the energy and temperature profiles, the systems reached thermal equilibrium rapidly and maintained stable fluctuations throughout the simulation. No atomic detachment, structural collapse, or irreversible rearrangement was observed. The catalysts maintained their structural integrity throughout the simulations, demonstrating excellent dynamic stability. Although the calculation of Gibbs free energy of formation and metal dissolution potential is beyond the scope of this work, the strongly negative binding energies ($E_{b}$ = −7.6 eV for TiC⊥BC_3_ and −1.6 eV for NiSe⊥BC_3_) suggest that the metal atoms are firmly anchored to the BC_3_ substrate, indicating good resistance against metal detachment and dissolution under operating conditions. We acknowledge that 4 ps AIMD simulations probe only the short-time dynamics. Longer time-scale (ns to μs) stability will be investigated in future work using classical molecular dynamics with machine-learned potentials.

### 3.2. Origins of HER Catalytic Activity

**Figure 4:**
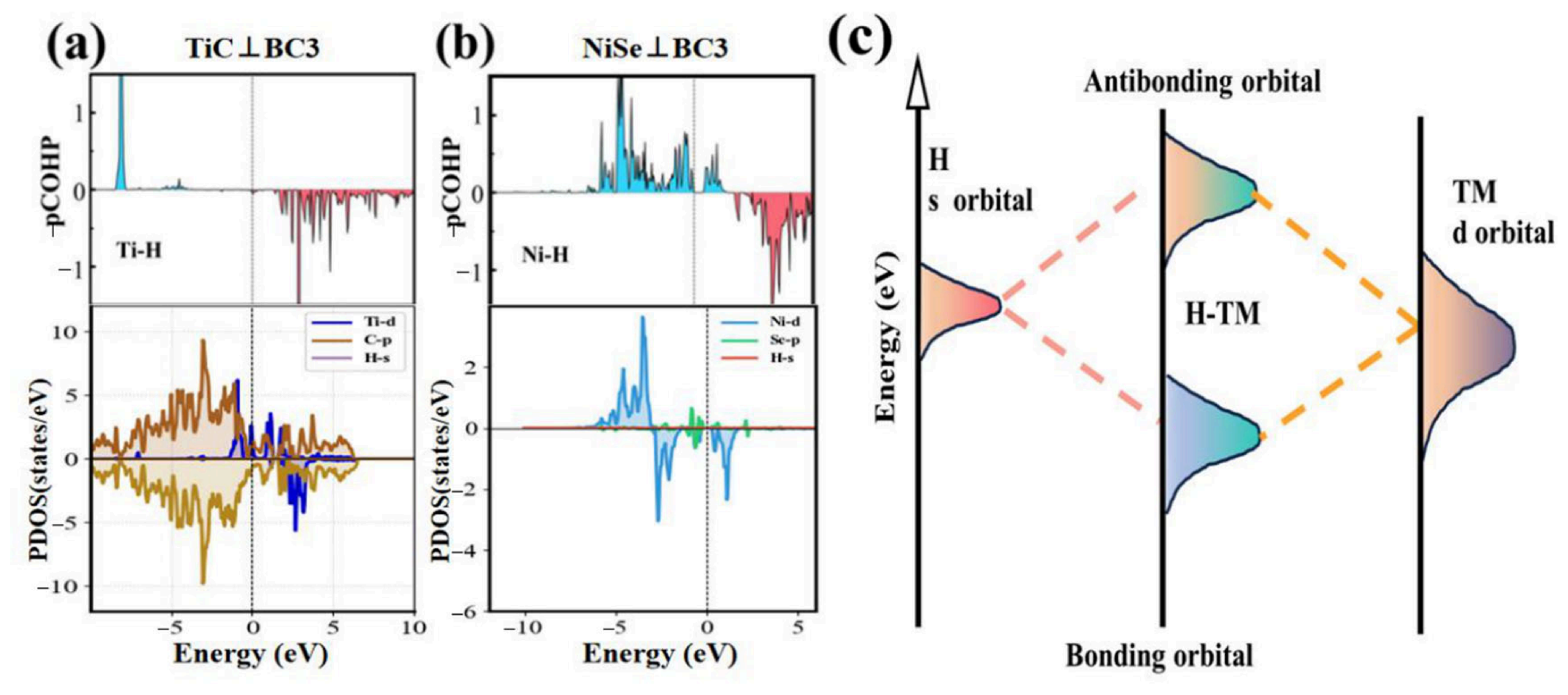
Orbital-resolved bonding analysis for hydrogen adsorption on (**a**) TiC⊥BC_3_ and (**b**) NiSe⊥BC_3_ based on PDOS and −pCOHP results. (**c**) Schematic illustration of orbital interactions between hydrogen s states and transition-metal d states. The colored curves correspond to the element-specific contributions identified in the legends of panels (**a**–**c**).

In dual-atom catalytic systems, metal–metal and metal–nonmetal interactions are strongly coupled. As a result, the origin of catalytic synergy cannot be explained by a single electronic descriptor. To elucidate the bonding characteristics at the dual-atom sites, projected density of states (PDOS) and crystal orbital Hamilton population (COHP) analyses were carried out (Figure 4a,b). The −pCOHP results reveal pronounced orbital overlap between transition metal (TM) and nonmetal (NM) atoms. This overlap provides clear electronic evidence for strong orbital hybridization and synergistic interactions within the dual-atom motifs. Further insights are obtained from the hydrogen–metal interaction analysis (Figure 4c). The electrons from the adsorbed hydrogen hybridize with the transition-metal orbitals, giving rise to both bonding and antibonding electronic states. Below the Fermi level, bonding states dominate, indicating that electrons preferentially occupy low-energy orbitals. This electronic configuration stabilizes the adsorption process. Notably, TiC⊥BC_3_ and NiSe⊥BC_3_ exhibit comparable −pCOHP integrated areas above and below the Fermi level. Such a balanced bonding–antibonding distribution suggests a moderate interaction strength. This balance is critical, as it enables efficient hydrogen adsorption while still allowing facile H_2_ desorption.

**Figure 5:**
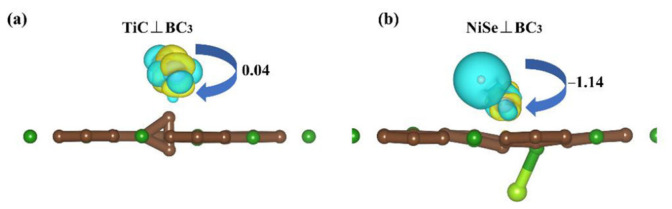
Charge-density redistribution induced by hydrogen adsorption on (**a**) TiC⊥BC_3_ and (**b**) NiSe⊥BC_3_. Yellow and light-blue regions indicate electron accumulation and depletion, respectively. Blue arrows indicate the direction of charge transfer, and the numerical values denote the Bader charge transferred from adsorbed H to the substrate (in e). Partial atoms at the lateral boundaries indicate the periodic continuation of the BC_3_ substrate.

Specifically, for TiC⊥BC_3_, the hybridization between Ti-3d and C-2p orbitals dominates the electronic coupling near the Fermi level. For NiSe⊥BC_3_, the analogous coupling occurs between Ni-3d and Se-4p orbitals. In both cases, the H-1s orbital further hybridizes with the TM-d states upon hydrogen adsorption, forming the bonding and antibonding pairs illustrated in Figure 4c. PDOS analyses further support this conclusion. The orbitals of TM, NM, and H atoms exhibit clear hybridization near the Fermi level, with most electronic states located below it. This feature reflects good electronic stability of the catalysts. At the same time, it ensures that hydrogen adsorption is neither too weak nor excessively strong. Charge redistribution upon hydrogen adsorption was visualized through differential charge density analysis (Figure 5a,b). Hydrogen preferentially binds to the transition metal sites. Electron accumulation and depletion regions clearly indicate substantial charge transfer between the TM atoms and the adsorbed hydrogen. While we acknowledge that atomic charges are not uniquely defined and different partitioning schemes may yield different numerical values [50], Bader charges provide a consistent and physically intuitive measure of charge redistribution within the same computational framework. Here, we use Bader charges primarily for comparative analysis between different TMNM⊥BC_3_ configurations rather than as absolute physical quantities. Bader charge analysis further quantifies this effect. Approximately 0.04 e and −0.19 e are transferred from the adsorbed hydrogen to the TiC⊥BC_3_ and NiSe⊥BC_3_ substrates, respectively. This moderate charge transfer helps establish a balance between adsorption stability and reaction kinetics. As a result, hydrogen can adsorb efficiently while remaining capable of recombination and desorption.

Compared with previously reported dual-atom catalysts on various substrates, such as Fe–Co/N-doped graphene [51] and Ni–Mo/C_3_N_4_ [52], our TiC⊥BC_3_ and NiSe⊥BC_3_ systems exhibit |${∆G}_{H*}$| values that are among the most favorable (0.03 and 0.09 eV, respectively). This comparison confirms that the anti-sandwich TM–NM architecture on BC_3_ is a competitive design strategy for HER.

Overall, the synergistic effect in dual-atom catalysts does not originate from a single structural or electronic factor. Instead, it arises from the cooperative interplay of orbital hybridization, charge redistribution, and bonding strength modulation. By tuning the electronic coupling between TM and NM atoms, the dual-atom sites achieve precise control over hydrogen adsorption energetics. This enables an optimal balance between thermodynamic stability and kinetic accessibility, ultimately leading to enhanced HER performance.

## 4. Conclusions

Using first-principles modeling, we explored the synergistic mechanisms of BC_3_-supported anti-sandwich dual-atom catalysts for HER. Screening a series of fourth-period transition-metal/nonmetal combinations identifies TiC⊥BC_3_ and NiSe⊥BC_3_ as highly active systems, exhibiting hydrogen adsorption free energies close to zero (~0.03 eV), which is comparable to or even closer to the thermoneutral condition than the benchmark Pt catalyst (with a typical |${∆G}_{H*}$| of ~0.09 eV from literature). Detailed electronic analyses demonstrate that the superior performance originates from synergistic orbital hybridization and charge redistribution within the dual-atom centers, providing fundamental insights into the design of advanced HER catalysts.

We note that the present calculations were performed under idealized conditions with implicit solvation. Explicit modeling of applied potential, pH, and electric double-layer effects remains challenging for large-scale DFT screening. Nevertheless, the thermodynamic ranking established here provides a solid baseline for identifying promising DAC candidates for future experimental and kinetic studies.

Binding energy calculations and ab initio molecular dynamics simulations confirm strong metal–substrate interactions. The dual-atom configurations maintain excellent structural integrity and resist atomic desorption under reaction conditions. Mechanistic analyses based on differential charge density, projected density of states (PDOS), and crystal orbital Hamilton population (COHP) reveal that the enhanced HER activity arises from cooperative orbital hybridization and charge redistribution between TM–H and TM–NM states. These findings provide a general electronic-structure framework for understanding synergistic effects in dual-atom catalysts and offer theoretical guidance for the rational design of high-performance anti-sandwich HER catalysts.

We hope that our theoretical predictions will stimulate experimental efforts to synthesize TiC⊥BC_3_ and NiSe⊥BC_3_ and to validate their HER performance under realistic operating conditions.

Developing a robust and transferable quantitative descriptor for dual-atom coupling synergy, beyond the conventional PDOS, COHP, and Bader charge analyses employed here, represents an important direction for future research in this field. We acknowledge that any atomic charge partitioning scheme has inherent limitations; nevertheless, a consistent descriptor—whether based on charge analysis, orbital overlap integrals, d-band characteristics, or other electronic metrics—could provide a practical guide for catalyst design if carefully benchmarked against experimental or high-level theoretical data.

## Data Availability

The raw data supporting the conclusions of this article will be made available by the authors on request.

## Supplementary Materials

The following supporting information can be downloaded from https://www.mdpi.com/article/10.3390/nano16150930/s1. Table S1: The Gibbs free energy of TMNM⊥BC_3_ systems; Table S2: The binding energies of TMNM⊥BC_3_ systems.
