# Supplementary material for: Electronic Synergy in Anti-Sandwich Dual-Atom Catalysts on BC3 for Efficient Hydrogen Evolution
Source: Nanomaterials (Basel). 2026 Jul 28;16(15):930. doi: 10.3390/nano16150930 (PMC13468414; doi:10.3390/nano16150930)
Supplement: Supplementary file 1 [file nanomaterials-16-00930-s001.zip › nanomaterials-4379883-supplementary.pdf]

Electronic Synergy in anti-sandwich Dual-Atom Catalysts on BC_3 for Efficient Hydrogen Evolution

Table S1. The Gibbs free energy of TMNM⊥BC_3 systems:

**Systems**	ΔG_{H*} **(eV)**
ScB⊥BC_3	**0.51**
ScC⊥BC_3	**1.40**
ScN⊥BC_3	**0.32**
ScSi⊥BC_3	**0.36**
ScP⊥BC_3	**0.20**
ScS⊥BC_3	**0.33**
ScAs⊥BC_3	**0.17**
ScSe⊥BC_3	**2.31**
TiB⊥BC_3	**1.43**
TiC⊥BC_3	**0.03**
TiN⊥BC_3	**1.81**
TiSi⊥BC_3	**2.30**
TiP⊥BC_3	**2.25**
TiAs⊥BC_3	**2.14**
TiSe⊥BC_3	**1.56**
VB⊥BC_3	**1.19**
VC⊥BC_3	**1.04**
VN⊥BC_3	**2.27**
VSi⊥BC_3	**0.83**
VP⊥BC_3	**1.60**
VS⊥BC_3	**2.25**
VAs⊥BC_3	**1.17**
VSe⊥BC_3	**2.36**
CrB⊥BC_3	**2.25**
CrC⊥BC_3	**2.28**
CrN⊥BC_3	**1.05**
CrSi⊥BC_3	**2.27**
CrP⊥BC_3	**2.27**
CrS⊥BC_3	**1.99**
CrAs⊥BC_3	**2.39**
MnB⊥BC_3	**1.19**
MnC⊥BC_3	**1.27**
MnN⊥BC_3	**1.03**
MnSi⊥BC_3	**1.05**
MnP⊥BC_3	**-2.33**
MnS⊥BC_3	**0.40**

MnAs⊥BC_3	**1.01**
MnSe⊥BC_3	**1.12**
MnTe⊥BC_3	**3.76**
FeB⊥BC_3	**0.90**
FeC⊥BC_3	**1.05**
FeN⊥BC_3	**1.06**
FeSi⊥BC_3	**0.69**
FeP⊥BC_3	**0.81**
FeS⊥BC_3	**1.38**
FeAs⊥BC_3	**0.73**
FeSe⊥BC_3	**1.19**
CoB⊥BC_3	**0.54**
CoC⊥BC_3	**0.75**
CoN⊥BC_3	**-0.22**
CoSi⊥BC_3	**0.68**
CoP⊥BC_3	**0.61**
CoS⊥BC_3	**0.94**
CoAs⊥BC_3	**0.57**
CoSe⊥BC_3	**0.84**
NiN⊥BC_3	**1.96**
NiSi⊥BC_3	**0.38**
NiP⊥BC_3	**1.86**
NiS⊥BC_3	**2.35**
NiAs⊥BC_3	**2.58**
NiSe⊥BC_3	**0.09**
NiTe⊥BC_3	**0.29**
CuB⊥BC_3	**0.49**
CuC⊥BC_3	**1.00**
CuN⊥BC_3	**0.39**
CuSi⊥BC_3	**0.57**
CuP⊥BC_3	**0.82**
CuS⊥BC_3	**0.44**
CuAs⊥BC_3	**0.64**
CuSe⊥BC_3	**0.33**
ZnB⊥BC_3	**-0.33**
ZnC⊥BC_3	**-0.24**
ZnN⊥BC_3	**-0.37**
ZnSi⊥BC_3	**-0.51**
ZnP⊥BC_3	**-0.61**
ZnS⊥BC_3	**-0.57**
ZnAs⊥BC_3	**-0.68**
ZnSe⊥BC_3	**-0.22**

Table S2. The binding energies of TMNM ⊥ BC_3 systems:

**Systems**	E_b**(eV)**
ScB ⊥ BC_3	**-9.5**
ScC ⊥ BC_3	**-10.9**
ScN ⊥ BC_3	**-9.1**
ScSi ⊥ BC_3	**-9.3**
ScP ⊥ BC_3	**-8.0**
ScS ⊥ BC_3	**-7.8**
ScAs ⊥ BC_3	**-7.5**
ScSe ⊥ BC_3	**-7.6**
TiB ⊥ BC_3	**-7.2**
TiC ⊥ BC_3	**-7.6**
TiN ⊥ BC_3	**-6.5**
TiSi ⊥ BC_3	**-7.0**
TiP ⊥ BC_3	**-5.8**
TiAs ⊥ BC_3	**-5.2**
TiSe ⊥ BC_3	**-5.2**
VB ⊥ BC_3	**-6.5**
VC ⊥ BC_3	**-7.3**
VN ⊥ BC_3	**-5.7**
VSi ⊥ BC_3	**-6.5**
VP ⊥ BC_3	**-5.1**
VS ⊥ BC_3	**-4.6**
VAs ⊥ BC_3	**-4.6**
VSe ⊥ BC_3	**-4.4**
CrB ⊥ BC_3	**-6.2**
CrC ⊥ BC_3	**-7.3**
CrN ⊥ BC_3	**-5.9**
CrSi ⊥ BC_3	**-6.2**
CrP ⊥ BC_3	**-4.9**
CrS ⊥ BC_3	**-4.3**
CrAs ⊥ BC_3	**-4.5**
CrSe ⊥ BC_3	**-4.3**
MnB ⊥ BC_3	**-7.1**
MnC ⊥ BC_3	**-8.3**
MnN ⊥ BC_3	**-6.7**
MnSi ⊥ BC_3	**-7.2**
MnP ⊥ BC_3	**-2.6**
MnS ⊥ BC_3	**-5.5**
MnAs ⊥ BC_3	**-5.4**
MnSe ⊥ BC_3	**-5.3**
MnTe ⊥ BC_3	**-4.8**

FeB⊥BC_3	**-4.7**
FeC⊥BC_3	**-5.0**
FeN⊥BC_3	**-4.3**
FeSi⊥BC_3	**-4.7**
FeP⊥BC_3	**-3.5**
FeS⊥BC_3	**-3.1**
FeAs⊥BC_3	**-3.0**
FeSe⊥BC_3	**-2.9**
CoB⊥BC_3	**-4.0**
CoC⊥BC_3	**-5.1**
CoN⊥BC_3	**-3.1**
CoSi⊥BC_3	**-4.3**
CoP⊥BC_3	**-2.9**
CoS⊥BC_3	**-2.4**
CoAs⊥BC_3	**-2.5**
CoSe⊥BC_3	**-2.1**
NiB⊥BC_3	**-3.6**
NiN⊥BC_3	**-3.2**
NiSi⊥BC_3	**-3.3**
NiP⊥BC_3	**-1.8**
NiS⊥BC_3	**-1.8**
NiAs⊥BC_3	**-2.1**
NiSe⊥BC_3	**-1.6**
NiTe⊥BC_3	**-1.3**
CuB⊥BC_3	**-3.5**
CuC⊥BC_3	**-4.7**
CuN⊥BC_3	**-3.4**
CuSi⊥BC_3	**-3.8**
CuP⊥BC_3	**-2.6**
CuS⊥BC_3	**-2.4**
CuAs⊥BC_3	**-2.1**
CuSe⊥BC_3	**-1.9**
ZnB⊥BC_3	**-3.8**
ZnC⊥BC_3	**-5.1**
ZnN⊥BC_3	**-3.3**
ZnSi⊥BC_3	**-3.8**
ZnP⊥BC_3	**-2.4**
ZnS⊥BC_3	**-2.1**
ZnAs⊥BC_3	**-1.9**
ZnSe⊥BC_3	**-1.9**
